# The ‘Densitometric Image Analysis Software’ and Its Application to Determine Stepwise Equilibrium Constants from Electrophoretic Mobility Shift Assays

**DOI:** 10.1371/journal.pone.0085146

**Published:** 2014-01-21

**Authors:** Liesbeth van Oeffelen, Eveline Peeters, Phu Nguyen Le Minh, Daniël Charlier

**Affiliations:** 1 Research group of Microbiology, Vrije Universiteit Brussel, Brussels, Belgium; 2 IMEC, Leuven, Belgium; Manchester University, United Kingdom

## Abstract

Current software applications for densitometric analysis, such as ImageJ, QuantityOne (BioRad) and the Intelligent or Advanced Quantifier (Bio Image) do not allow to take the non-linearity of autoradiographic films into account during calibration. As a consequence, quantification of autoradiographs is often regarded as problematic, and phosphorimaging is the preferred alternative. However, the non-linear behaviour of autoradiographs can be described mathematically, so it can be accounted for. Therefore, the ‘Densitometric Image Analysis Software’ has been developed, which allows to quantify electrophoretic bands in autoradiographs, as well as in gels and phosphorimages, while providing optimized band selection support to the user. Moreover, the program can determine protein-DNA binding constants from Electrophoretic Mobility Shift Assays (EMSAs). For this purpose, the software calculates a chosen stepwise equilibrium constant for each migration lane within the EMSA, and estimates the errors due to non-uniformity of the background noise, smear caused by complex dissociation or denaturation of double-stranded DNA, and technical errors such as pipetting inaccuracies. Thereby, the program helps the user to optimize experimental parameters and to choose the best lanes for estimating an average equilibrium constant. This process can reduce the inaccuracy of equilibrium constants from the usual factor of 2 to about 20%, which is particularly useful when determining position weight matrices and cooperative binding constants to predict genomic binding sites. The MATLAB source code, platform-dependent software and installation instructions are available via the website http://micr.vub.ac.be.

## Introduction

To calibrate an autoradiograph, a calibrated step tablet should be scanned, preferrably together with the autoradiographic film to be analyzed, as the sensitivity of the scanner may change over time. Based on the relation between the average gray values of the steps in the step tablet and their optical densities, the measured gray values in the autoradiograph can be mapped to optical densities by curve-fitting or linear interpolation. However, the relation between optical density and concentration of radioactive material is not linear, and therefore, a second mapping should be performed, from optical densities to relative concentrations 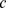


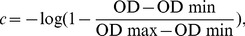
(1)with 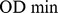 and 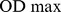 the minimum and maximum OD's of the autoradiographic film, i.e., the OD after development of an unexposed film and a film exposed to broad daylight [1]. Current software applications for densitometric analysis, such as ImageJ, QuantityOne (BioRad) and the Intelligent or Advanced Quantifier (Bio Image) do not offer the possibility of a second mapping and/or a fit to the curve described by equation 1, and therefore display the same non-linearity as the film. The ‘Densitometric Image Analysis Software’ has been developed to solve this issue. Moreover, apart from measuring electrophoretic band intensities in autoradiographs, gels and phosphorimages, this software also allows to determine binding constants from Electrophoretic Mobility Shift Assays (EMSAs).

The EMSA assay is the main technique used to study protein-DNA interactions [2], [3]. In its most sensitive form, this technique consists of incubating radioactively labeled DNA with one or more species of DNA-binding proteins, and separating the different complexes by gel electrophoresis, after which they can be visualized by means of autoradiography or phosphorimaging. The main advantage of EMSAs compared to other techniques such as filter binding assays and surface plasmon resonance, is that complexes with different migration velocities (stoichiometries, …) can be separated and their ratios determined.

EMSAs are often interpreted qualitatively: to establish whether or not a given protein binds a certain DNA fragment or oligonucleotide duplex, whether a co-factor affects DNA binding, or whether the binding affinity of a protein is influenced by another DNA-binding protein, resulting in a cooperative or competitive effect. However, protein-DNA binding in EMSAs can also be quantified. A quantitative study yields a more detailed view on the protein-DNA interaction and its involvement in the cognate DNA transactions (transcription, replication,…). Moreover, it allows measurements of position weight matrices [4], [5] and cooperative binding constants that can be used to predict new binding sites in the genome [6].

One way to quantify protein-DNA binding is by using a curve-fitting approach [4], [7]–[9]. However, curve-fitting only provides binding parameter values, no standard deviations. Therefore, we prefer to determine a stepwise equilibrium constants for each lane 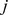 as follows:
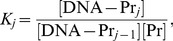
(2)with Pr representing the protein, and we calculate an average value and a standard deviation.

We show that, by carefully taking into account the different error sources, this allows us to determine stepwise equilibrium constants with uncertainties around 20% instead of the usual factor of about 2 [10]. As such, accurate position weight matrices [5] and cooperative binding constants [11] could be determined, which can further be used to predict genomic binding sites.

## Methods

This section describes the entire program, how cooperative binding constants and their standard deviations can be determined, and a method to simulate smear caused by the dissociation of protein-DNA complexes.

### Uploading the data and pre-processing

First, the program interactively loads an Excel file with a sheet named ‘Data’, containing the experimental data of one or more autoradiographs, gels and/or phosphorimages. An example file can be downloaded from http://micr.vub.ac.be. For each experiment, the ‘Data’ sheet contains the file name of the image, the number of bands in this image, in case of an autoradiograph the minimum and maximum attainable optical densities of the autoradiographic film, and, in case of an autoradiograph or gel, the file name of the image of a calibrated step tablet and the optical density values of this step tablet. If the user wants to determine binding constants from an EMSA, he also needs to provide the molar weight and concentration of the protein, the protein volumes added to the different lanes, and the total sample volume. This allows the program to determine concentrations. If the user only wants to obtain band intensities, he can either provide the same additional information, or only indicate the number of lanes in the image. The user can then select an image, of which the gray values are filtered with a 5 by 5 pixels running average filter to reduce the grainyness of the picture and measurement noise of the scanner. The resulting values are mapped to optical densities based on the measured gray values of the step tablet and using linear interpolation. In case of a phosphorimage or gel image, count values and OD's resp. represent relative concentrations. In case of an autoradiograph, optical densities (OD) are mapped to relative concentrations 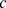 of radioactive material, using Equation 1.

In our research, Kodak Biomax MR films have been used with minimum and maximum OD's determined as 0.06 and 1.6, respectively. Autoradiographs and gels were scanned with a Microtek Bio-5000 scanner, with a bit depth of 16 bits and a resolution of 600 dpi. In each EMSA scan, the DNA band is placed at the bottom of the image, and within the program, this band is referred to as band 1.

**Figure 1:**
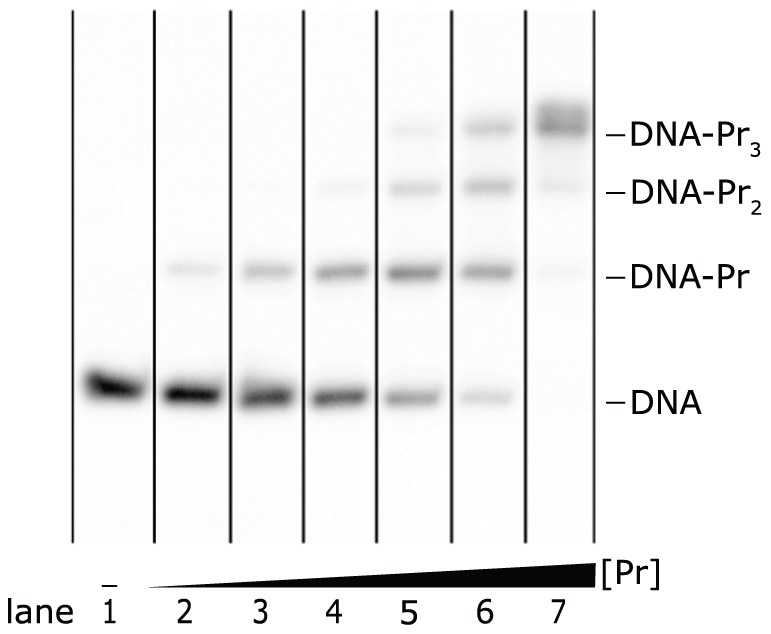
Autoradiograph of an EMSA. Dimeric Ss-LrpB protein binds to the control region of the cognate gene, containing three binding sites for the regulator. The Ss-LrpB protein concentration 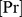 varies stepwise from 0 (lane 1) to 287 nM (lane 7), while the DNA concentration remains constant at about 0.1 nM. The positions of the free DNA and the various protein-DNA complexes with a different stoichiometry are indicated. Vertical lines have been drawn automatically by the program to separate the different migration lanes.

The software displays a calibrated version of the original image, i.e., with gray values proportional to relative concentrations. An example is shown in figure 1. Hence, relative concentrations correspond to intensities in this image, and the terms ‘relative concentration’ and ‘intensity’ will further be used interchangeably.

**Figure 2:**
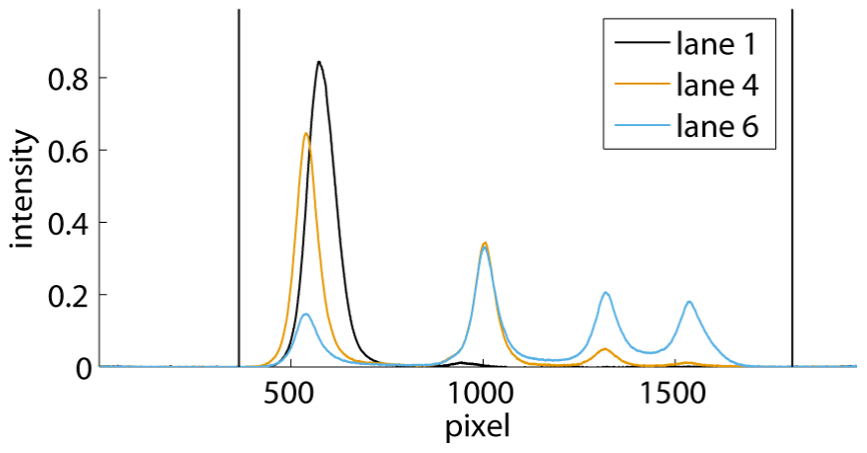
Average lane intensities. Curves are shown for lanes 1, 4 and 6 from the autoradiograph of the EMSA in figure 1. The traces from left to right correspond to horizontal intensity averages over a lane from the bottom to the top of the autoradiograph. The parts at the left of the first vertical line and at the right of the second line have been used to estimate and subtract the background.

If necessary, the user can remove artifacts, such as scratches or spots that occurred during development, by selecting each time two opposite corners of a rectangle covering the artifact area, which is excluded from further analysis. Subsequently, the boundaries separating the different migration lanes can be detected automatically by the program, or selected manually by the user. The program then plots the average lane intensities of all the lanes, similarly to figure 2, and the user selects a background subtraction method. Preferably, the double-sided subtraction method is chosen, in which the user selects the part at the far left and right where the curves are approximately flat. For each lane, the program subtracts the background that is estimated using the average intensities in that lane at the left of the first line and at the right of the second line, and assuming a linear dependence in between. Alternatively, the user can choose between a single-sided subtraction, or select a part of the background in the image, in case that not all lanes reach the background level at the left and/or the right.

### Interpretation of an EMSA: DNA, protein-DNA complexes, single-stranded DNA, and smear

**Figure 3:**
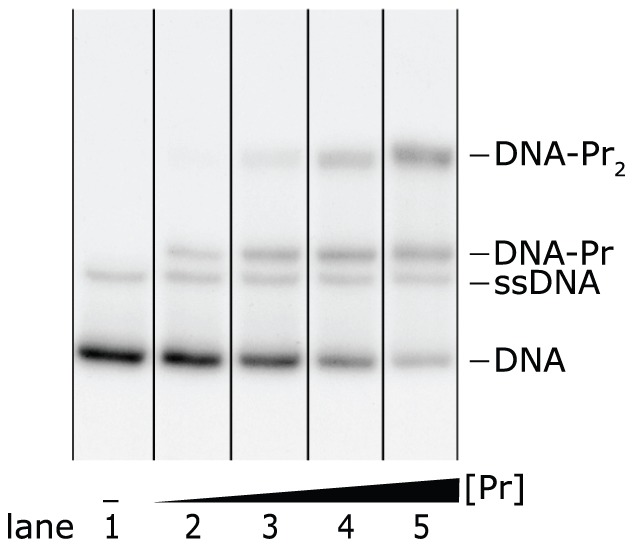
Autoradiograph of an EMSA showing a distinct band of single-stranded DNA (ssDNA). Four different concentrations of dimeric Ss-LrpB were allowed to bind to a DNA fragment bearing two binding sites for the regulator. The positions of the free DNA, ssDNA and the two protein-DNA complexes with different stoichiometries are indicated. Vertical lines separate the different migration lanes identified by the program.

An EMSA typically contains several bands, with an amount of smear in between. While bands with intensities depending on the protein concentration can be attributed to DNA and protein-DNA complexes, with ‘DNA’ referring to double-stranded DNA, bands with virtually constant intensities over the lanes represent a contamination or single-stranded DNA (ssDNA). An ssDNA band as seen in figure 3 is generated by dissociation of short DNA molecules at very low concentrations, and often migrates more slowly than the corresponding DNA. The fact that its intensity remains virtually constant over the lanes indicates that the binding rate of ssDNA to form DNA is so low that, while an equilibrium is formed between DNA and the protein-DNA complex during incubation, this is not the case for ssDNA and DNA. Hence, an ssDNA band represents ssDNA formed before electrophoresis, and therefore should be removed from our analysis together with any contaminating bands as described later on.

**Figure 4:**
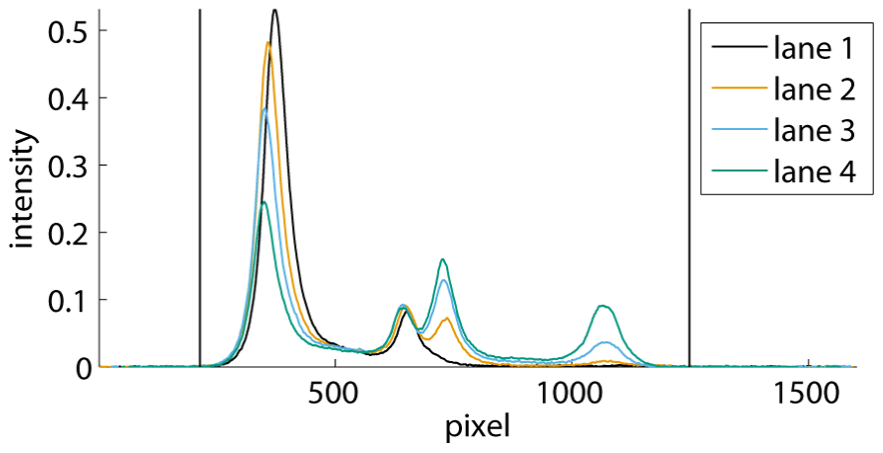
Average lane intensities of lanes 1 to 4 from the EMSA in figure 3. ssDNA smear is observed as a widening of the first peak towards the right side. The smear in lane 1, in which no protein was added, is retrieved between the unbound DNA and the ssDNA bands.

Smear on the other hand is often caused by complex dissociation. However, in most EMSAs we also noticed a significant amount of smear in the lane with no protein added. This can be attributed to the formation of (partially) single-stranded DNA during gel electrophoresis: if there is a distinct ssDNA band, the smear is percieved between this band and the DNA band, as shown in figure 4. Moreover, the amount of smear increases with the amount of DNA. Hence, ssDNA smear originates from DNA during electrophoresis, and therefore should be accounted for in the DNA band, as described in the next section.

### Selecting the boundaries between the different bands in the presence of smear

**Figure 5:**
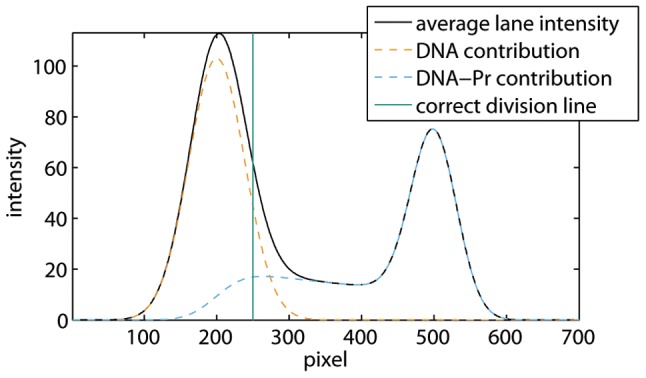
Simulation of dissociating protein-DNA complexes. The average lane intensity and the contributions due to DNA and protein-DNA complexes are shown together with the correct division line. The simulation was performed assuming equal initial DNA and protein-DNA concentrations, and a diffusion coefficient of DNA that is 1.5 times the diffusion coefficient of the protein-DNA complex, which is a realistic approximation. As both the concentrations and the diffusion coefficients are determined up to a constant factor, the units in this figure are arbitrary.

Figure 5 shows a simulation of dissociating protein-DNA complexes obtained with the method discussed at the end of this Methods section. The vertical line in this figure corresponds to the division line that yields correct band intensities: the integrated average lane intensities at the left and the right of this line equal the integrated DNA and protein-DNA contributions, respectively. As these individual contributions are unknown in an experiment, the correct division line cannot be determined exactly in the presence of smear. Hence, the program allows the user to select two vertical lines representing an upper and a lower bound of the space in which the actual division line should be located. The smear between these bounds cannot be attributed to the upper or lower band with certainty. Therefore, our program assigns half the amount of smear to each of the two bands, and estimates the error due to smear as will be described later on. In this section, we first explain how to select the upper and lower bounds, and illustrate our approach on the simulation, thereby demonstrating that the correct division line is retrieved in between. Second, we apply this method to the EMSA from figure 1.

**Figure 6:**
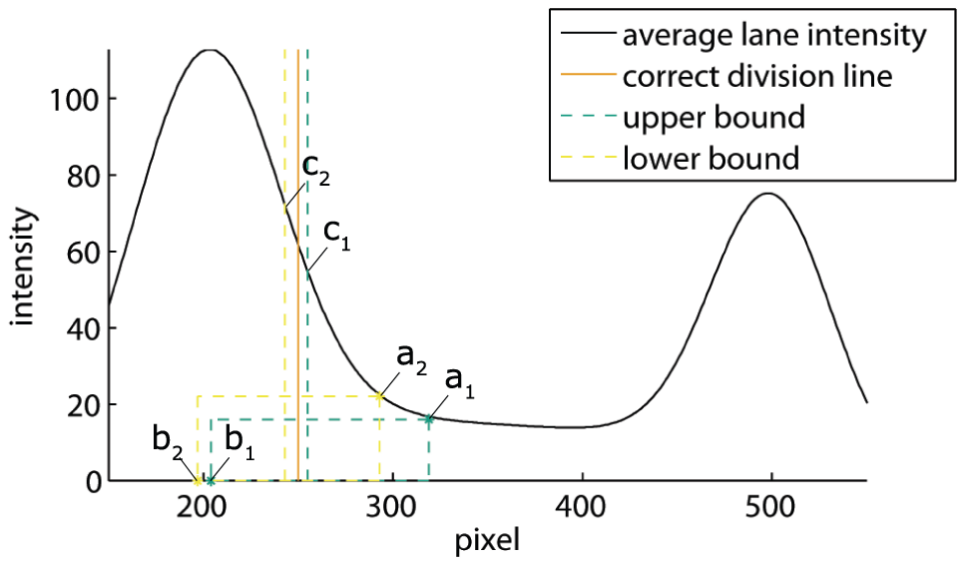
Selection of band boundaries. The simulated average lane intensity is shown together with the correct division line, and the upper and lower bounds determined in such a way that the area under the average lane intensity curve between points 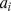 and 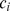 equals the area of the rectangle with corners 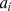 and 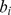, indicated by the user.

To determine an upper bound, the user first selects a point on the average lane intensity curve within the smear close to the DNA band, such as point 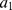 in figure 6. Then he selects a second point 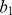, vertically aligned with the peak of the DNA band, and horizontally at an overestimate of the ssDNA smear, which is zero in the present case. The area of the rectangle drawn with these two points yields an underestimate of the smear due to protein-DNA dissociation at the left of point 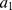. Therefore, the division line through 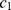, obtained by shifting the vertical line through 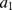 to the left until the area removed from the DNA band equals the underestimated smear, is an upper bound of the correct division line. This bound is automatically drawn by the program.

A lower bound can be determined in a similar way, with point 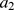 located where the average lane intensity reaches an overestimate of the smear, and with 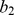 slightly at the left of the DNA peak and at an underestimate of the ssDNA smear, which is again zero in this case. The reason for selecting the second point more to the left is that, due to dissociation smear, the DNA peak is shifted slightly to the right as can be observed in figure 5.

In general, the contribution of ssDNA to the smear can be estimated making use of the fact that ssDNA smear is proportional to the amount of dsDNA. The inaccuracy of this estimate should be taken into account to determine the over- and underestimate of this smear.

In the absence of smear or in the case of overlapping bands, the user can select division lines completely manually, by positioning the first point at the place where the division line should occur, and the second somewhere at the right of this point.

**Figure 7:**
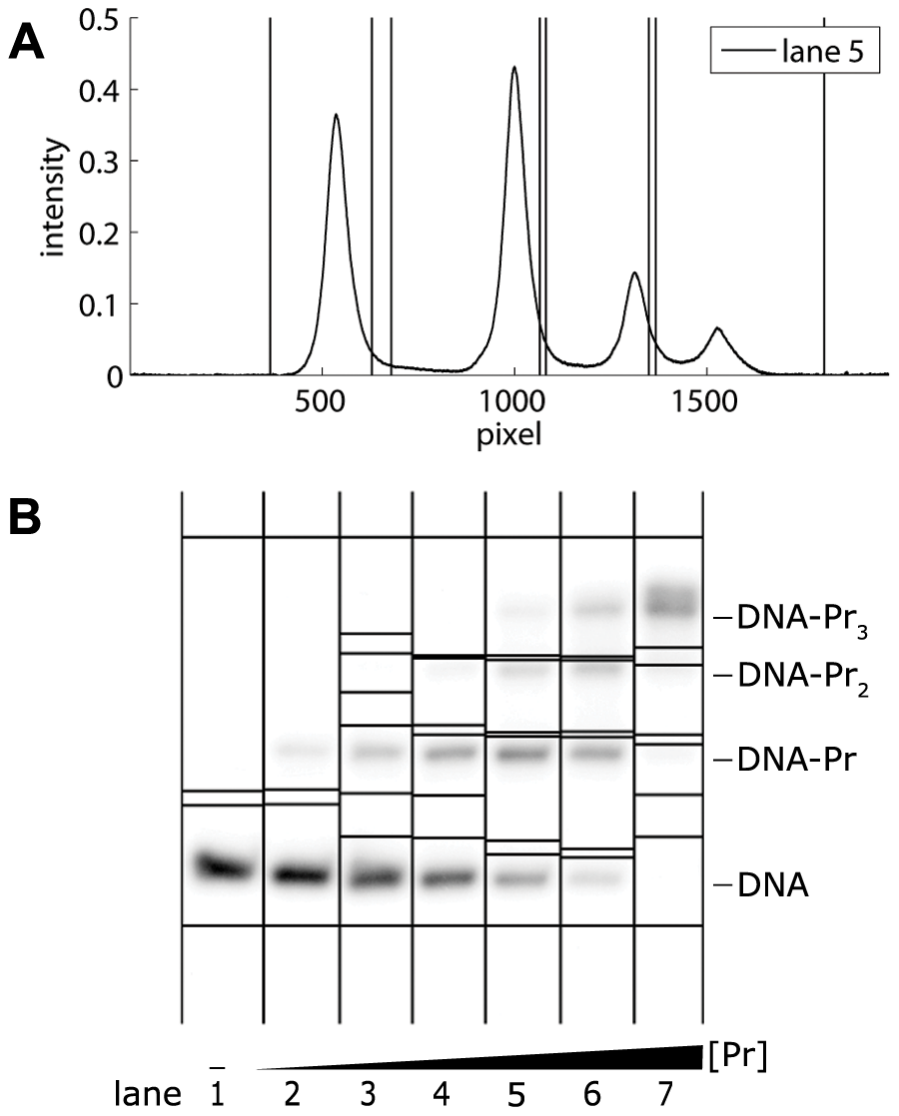
Band selection in the EMSA of figure 1. **A** Average lane intensity and band selection performed in lane 5. **B** Autoradiograph of figure 1 with horizontal lines representing band boundaries. These boundaries are transferred to the autoradiograph each time a pair of upper and lower band boundaries is selected within a lane.

Figure 7 illustrates the results of the band selection method on the EMSA from figure 1. The bounds selected in figure 7A are automatically transferred to the EMSA in figure 7B. The first and the last vertical lines in figure 7A (and the corresponding horizontal lines in figure 7B) represent the background subtraction lines, which also function as band boundaries of the first and the last band.

### Removing an ssDNA or a contaminating band

**Figure 8:**
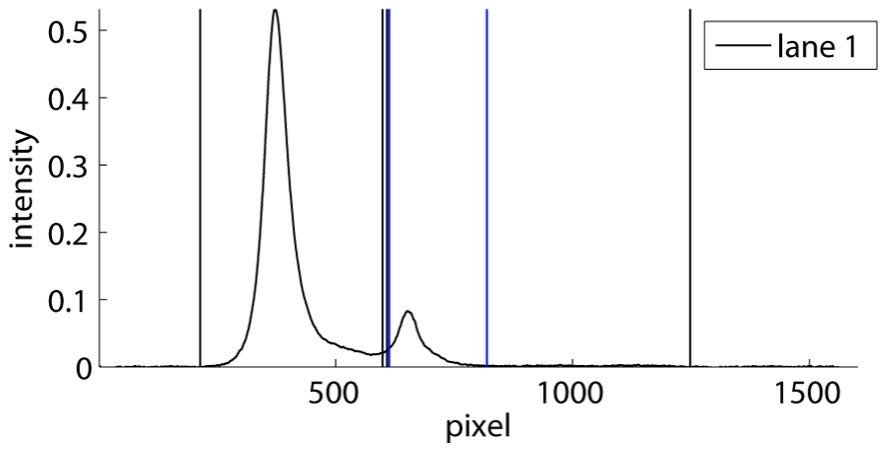
Example of ssDNA band subtraction. The average lane intensity curve of lane 1 from the EMSA in figure 3 is shown together with black band boundaries and blue lines that indicate the ssDNA band. This band is selected within and thus also subtracted from the protein-DNA band.

Our program offers two possibilities to remove an ssDNA band: (i) its intensity measured in one lane can be subtracted from all the lanes, or (ii) it can be literally removed from the EMSA. The first option can be applied in the most general case that the ssDNA band overlaps the DNA or the protein-DNA band, or that this band lies within a significant amount of smear. For this purpose, the user selects the lane in which the ssDNA band can be distinguished best, usually a lane without protein added, and draws two vertical lines to select the ssDNA band within a band or in the smear between two bands, as shown in figure 8. The integrated intensity over the selected area is then subtracted from this band or smear in all the lanes.

If the ssDNA band does not overlap the DNA or the protein-DNA band and if the smear between the DNA and protein-DNA bands is comparatively low, the second option can be used as well. In this case, the user again selects two lines in a figure of average lane intensities, between which the program sets the intensities to zero in all the lanes. These two strategies of band removal and subtraction can be repeated for any contaminating band.

### Determination of band intensities

**Figure 9:**
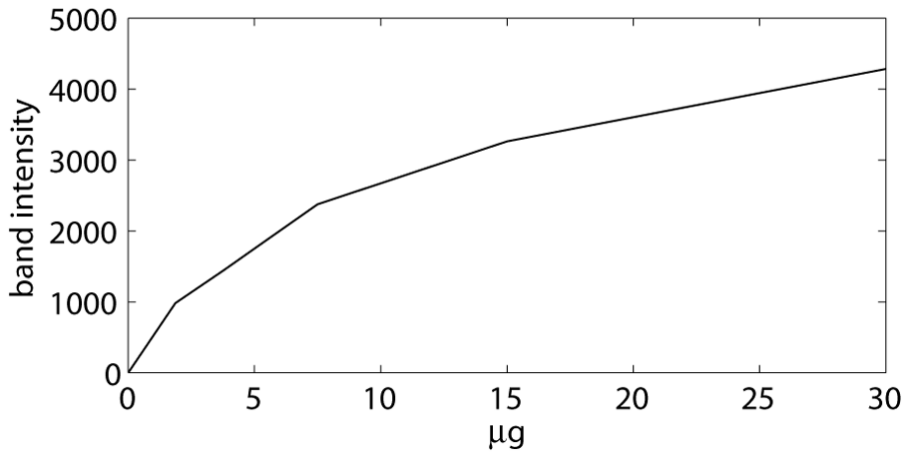
Relation between amount of protein and band intensity for a stained protein gel.

In the previous sections, band selection and subtraction have been illustrated with EMSAs. These methods can also be used with for example protein gels. In this case, the user typically wants to determine band intensities, while in the case of an EMSA, the user may also want to calculate a binding constant. The software provides both options. In the first case, band and smear intensities are written to an excel file. The second option is discussed in the next paragraph. It must however be noted that the staining process in gels may yield a non-linear relation between amount of protein and band intensity, as illustrated in figure 9. Moreover, this relation seems irreproducible, meaning that the concentration of a sample can only be determined if this sample is loaded on the same gel with a series of known concentrations.

### Determination of a stepwise equilibrium constant

**Figure 10:**
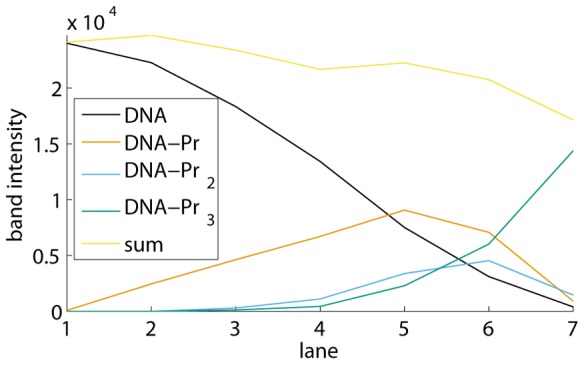
Band intensities of free DNA, the different protein-DNA complexes, and their sum. The values shown are those of the EMSA in figures 1 and 7B.

After having performed all necessary band removals and subtractions, the user can select a K-value, a stepwise equilibrium constant which may be 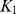, 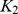 or 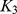 for the example shown in figure 7. At this point, the program generates a figure with the integrated band intensities across the lanes and their sum, as in figure 10. As such, the user obtains an indication of pipetting inaccuracies.

**Figure 11:**
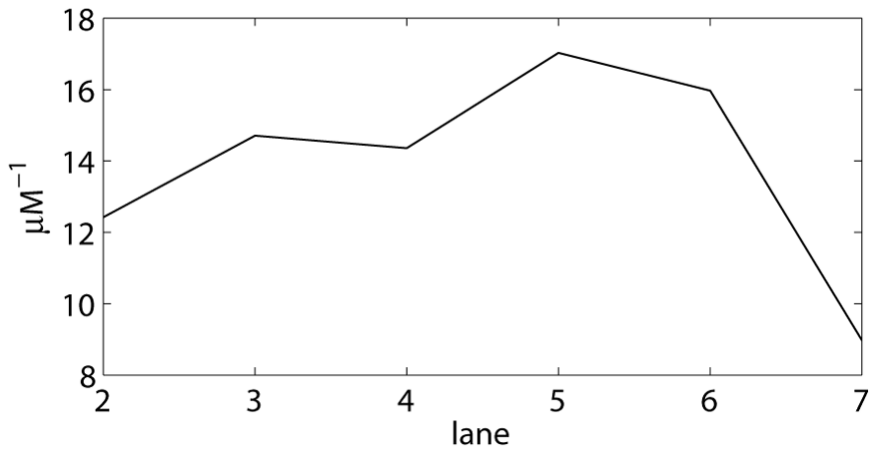
Stepwise equilibrium constant 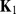. Values are calculated based on the band intensities shown in figure 10.

The chosen stepwise equilibrium constant is calculated as
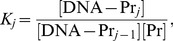
(3)with 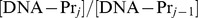 corresponding to the ratio of 2 band intensities, and 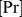 the equilibrium protein concentration. In our program, this concentration is assumed equal to the initially added concentration - an approximation which is only valid if the protein concentrations used are much higher than the DNA concentration. Therefore, the DNA concentrations in our experiments are typically about 0.1 nM, while the protein concentrations range between 1 nM and a few 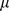
M. The K-values calculated in this manner are automatically plotted across the lanes, as shown in figure 11 for 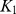.

### Estimating errors and selecting the best lanes

To further enable the user to select the best lanes from the EMSA for the determination of an average stepwise equilibrium constant, the program estimates the different error contributions for each lane. In the case that the DNA fragment contains a single binding site, and that 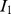 and 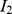 are the band intensities of unbound and bound DNA in lane 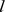, the measured association constant equals:
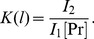
(4)This value is influenced by three different error sources: a non-uniform background, smear, and technical errors. Therefore, the measured 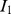 and 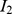 can be written as
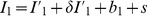
(5)and
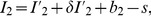
(6)with 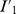 and 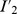 the correct intensities; 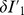 and 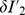 the errors due to technical inaccuracies; 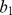 and 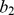 the contributions of background noise; and 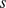 the amount of smear that is assigned to the wrong band, counted positive if that band is the DNA band and negative if it is the protein-DNA band. As a result, 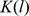 can be written as:
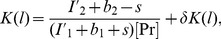
(7)with 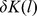 the contribution due to 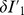 and 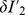.

A first order Taylor series expansion yields:
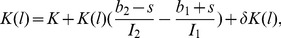
(8)with 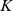 the correct association constant. Hence, the errors due to background noise and smear are respectively:
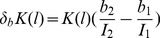
(9)and
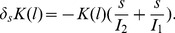
(10)


The three error sources may contribute in both a random and a systematic fashion. Random errors can be reduced by averaging over different lanes, and the standard error of the mean 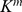, namely 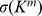, yields an uncertainty measure. Systematic errors, however, cannot be reduced by averaging, and therefore should be avoided as much as possible. The way to achieve this is described in the results section.

Here, we postulate that technical errors as well as the errors due to a non-uniform background are random, while the smear error is assumed to be systematic. The rationale behind this reasoning lies in the the fact that the amount of smear between the user-selected upper and lower bounds is on average not symetrically distributed around the real division line. More specifically, we hypothesize that both the errors due to a non-uniform background and smear are randomly distributed between a minimum and a maximum value, but that smear errors are correlated over the lanes, resulting in a systematic error, while background errors are not.

To estimate the maximum value for 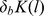, the program performs the following steps for both the left (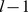) and the right (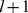) lane, if both are available: it calculates the difference in background, integrates this between the beginning and the end of each band, yielding 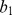 and 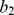, and applies equation 9 to obtain an error estimate. The maximum of the absolute values of the error estimates then serves as an estimate of the maximum error 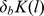. Assuming that the error is randomly distributed between 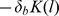 and 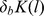, the standard deviation of this error can be calculated as
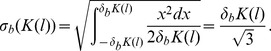
(11)


To estimate the maximum smear error 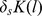, we substitute 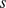 in equation 10 by plus or minus half the amount of smear between the two bands. This means that the maximum positive and negative smear errors are calculated by taking the smear between the bands into account first in the protein-DNA band and then in the DNA band. The standard deviation of the measured K-value due to smear, 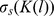, can then be calculated in a similar way as 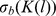:
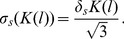
(12)


In the case that the DNA has more than one binding site, 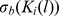 can be determined in an analogous way as 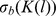 described before, while for 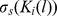, not only the smear between the two bands has to be considered as in equation 10, but also the smear in front of the first band and behind the second band. For this purpose, the maximum errors and standard deviations of the 3 smear contributions are determined separately, and, assuming that these are independent, 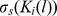 is calculated as the square root of the sum of the variances.

**Figure 12:**
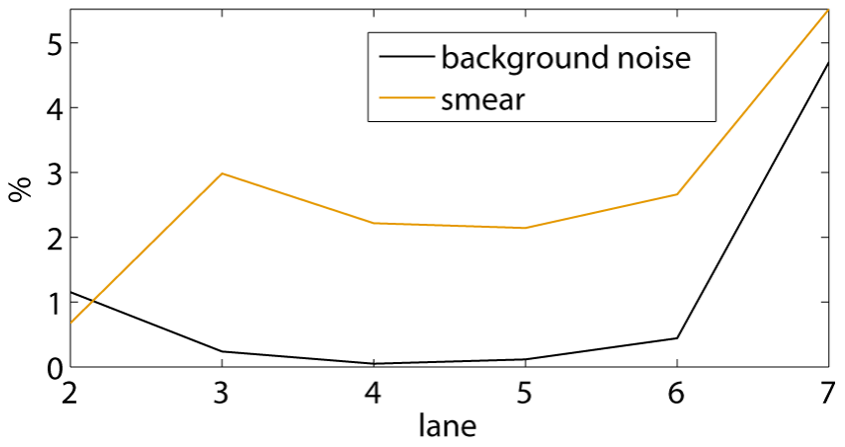
Relative standard deviations due to a non-uniform background and smear. These values represent estimated errors on 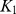 in figure 11.

Both the relative standard deviations 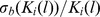 and 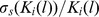 are automatically plotted by the program, as in figure 12. Together with figures 10 and 11, this allows the user to select the best lanes from the EMSA for the determination of an average value. In the example shown, lanes 2 until 6 are selected: no obvious pipetting inaccuracies occur in figure 10, while the practically invisible amount of DNA in lane 7 results in larger background noise and smear errors, which may explain the different K-value in lane 7.

### Estimating errors on the average K-value

The standard deviation of the average K-value due to non-uniform background noise can be calculated as:
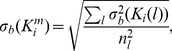
(13)with 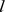 running over the user-selected lanes and 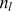 its number. The standard deviation of the systematic error of the average K-value 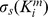 equals
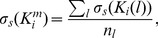
(14)since the errors are assumed correlated over the lanes. Hence, the total standard deviation of the average K-value is
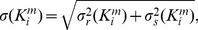
(15)with 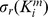 the contribution of random errors, which is the measured standard deviation, representing the contributions of a non-uniform background 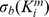 and technical errors such as pipetting inaccuracies 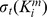:
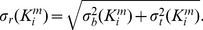
(16)


Finally, the average K-value, its standard deviation, the standard deviations due to background noise and smear, and the resulting total standard deviation, which includes systematic smear errors, are displayed on the screen and automatically written to a sheet named ‘Results’ in the original data file. The same is done for the normalized values 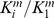 and its standard deviation. The calculation of this standard deviation and its use are described in the next section.

### From stepwise equilibrium constants to a cooperative binding constant

Here, we demonstrate how cooperative binding constants, i.e., binding constants corresponding to protein-protein interactions, can be determined in the case of homotypic cooperative protein binding at two non-overlapping sites. We name the cooperative binding constant 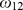, the sites Box1 and Box2, and their intrinsic binding affinities 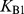 and 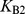, which can be determined in independent EMSAs with a DNA fragment containing only Box1 or Box2. An EMSA with the complete construct then yields the following value for 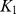:
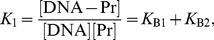
(17)as the protein-DNA band consists of both DNA bound at Box1 and DNA bound at Box2. 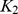 on the other hand can be determined by substituting
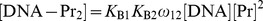
(18)and equation 17 in
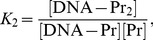
(19)which yields:
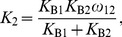
(20)and after normalization with 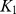:
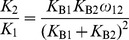
(21)

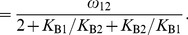
(22)Hence,
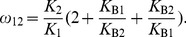
(23)


Equation 22 is very similar to the one used by Fried and Daugherty [12]. The only difference is that this group determines 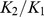 lane per lane, while we first determine an average 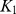 and 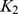, in general using different lanes, and then calculate their ratio. As a consequence, our approach does not require all three bands to be present simultaneously in one lane.

On the other hand, it is also possible to compare cooperative binding constants of experiments in which the same two non-overlapping binding sites are separated by different distances and/or sequences. In this case, we do not need absolute values for 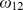, and 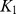 can serve as a reference as discussed in the results section. Hence, 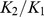 in equation 22 can be used as a relative measure of 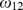, and therefore allows relative comparison of cooperativity.

To estimate the standard deviation of 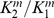, we use a Taylor series expansion:
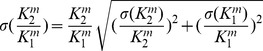
(24)The standard deviation of 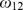 can be estimated in a similar way as follows:
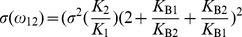


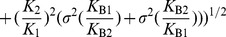
(25)


### Simulating the dissociation of protein-DNA complexes

A numerical method to simulate complex dissociation has been described by Cann [13]. Here, we use a simpler model in which the association of DNA and proteins during electrophoresis is neglected, as proteins and DNA lack physical proximity and optimal buffer conditions. Suppose that 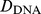 and 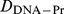 are the diffusion coefficients of the DNA and the protein-DNA complex, respectively, and that 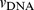 and 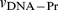 are their velocities due to electrophoresis, which is performed during a time span 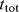. The protein-DNA complexes that dissociate between 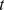 and 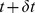 then result in a Gaussian distribution with a mean of
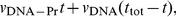
(26)and a standard deviation of
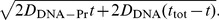
(27)The smear can therefore approximately be modelled as a convolution of a Gaussian with the amount of protein-DNA complex that dissociates as a function of time. This explains our band selection procedure: the amount that dissociates is proportional to the amount that is left, and hence decreases toward the protein-DNA band.

## Results and Discussion

In this section, we discuss the possibilities to deal with the different error sources observed when determining stepwise equilibrium constants, and validate our method.

### Dealing with errors

As mentioned in the Methods section, the three major error sources, i.e., non-uniformity of the background noise, smear, and technical errors, can contribute in both a random and a systematic way. In this part, we first describe the procedures to reduce the systematic errors, and then the random ones.

Systematic non-linear background variations can be detected in a figure of average lane intensities (such as figure 2) and may be caused by background light. Alternatively, the scanner may be responsible for non-linear background variations. For example, with the Bio-Rad GS-800 scanner we observed relatively high background variations in the measured gray values compared to the Microtek Bio-5000 scanner. However, after mapping to optical densities and/or relative concentrations, the effect of these variations becomes hardly visible: the sensitivity of optical densities and relative concentrations to background variations in light areas of the gel or film is very low. On the other hand, systematic smear errors arise if the amount of smear between the user-selected upper and lower bounds is on average not symmetrically distributed around the correct division line. However, these errors cannot easily be avoided as reducing smear requires the identification of factors that contribute to the stability of protein-DNA complexes during electrophoresis [12]. Nevertheless, careful choices of upper and lower bounds generally lead to acceptable error estimates. Furthermore, a major technical issue may arise when experiments are not conducted in parallel: after protein purification, the fraction of actively binding protein decreases over time. Therefore, experiments performed over a relatively long time span cannot be compared directly. First, they should be normalized, for example, using the binding constant of a reference sequence, which is measured in parallel every time new experiments are conducted. Other technical errors such as pipetting inaccuracies can be avoided by excluding the lanes in which they are observed in figure 10, or, if a visible error occurred in making different protein dilutions, by using only lanes with different amounts of the same protein dilution to determine the K-values of both the considered EMSA and the reference EMSA. Inaccurate ssDNA band subtraction, which is also considered a technical error, is difficult to avoid. Therefore, even though the magnitude of the inaccuracy can be estimated by repeating the subtraction, EMSAs without an ssDNA band should be given preference.

Random errors are characterized by the standard deviation of the average K-value over the selected lanes. To avoid underestimation of this standard deviation by selection of lanes in which the K-values are coincidentally close, we advise the user to select at least 3 lanes for the calculation of each stepwise equilibrium constant. According to equation 16, the random errors consist of contributions of a non-uniform background and technical errors. In reality, smear errors may also contribute, but we made the safe assumption that smear errors are correlated, and thus result in a systematic error, to avoid underestimating their effect on the total standard deviation of the average K-value that includes systematic errors. The contribution of the non-uniform background can be estimated separately. If this contribution is relatively high, the simplest and most effective solution is to expose a new film to the gel for a longer time period as this yields a better signal-to-noise ratio. The contribution of technical errors can be derived from equation 16, but may be overestimated as a consequence of random smear errors. In any case, both technical and smear errors cannot easily be avoided.

The user should be aware of a last type of errors, being modelling errors. A main example is the assumption that all the protein added to the reaction adopts an actively binding oligomeric state. When this is not the case, the user may for example observe a K-value that depends on the protein concentration.

### Validation

To check if no important error sources have been omitted in our method to determine stepwise equilibrium constants, we examined if differences between estimated stepwise equilibrium constants from repeated experiments are comparable to their standard deviations within the extensive experimental dataset from Peeters et al. [11], and we checked if this is also the case for repeated experiments with Sa-LysM [5]. The raw datasets used in these references, containing inputs and outputs of the Densitometric Image Analysis Software, are available at http://micr.vub.ac.be. Before normalization, the differences in stepwise equilibrium constants can be a factor of 2 or even more while the relative standard deviations are about 15%. After normalization, stepwise equilibrium constants differ on average by approximately 20%, while the estimated relative standard deviations are also about 20%. This indicates that indeed no important error sources have been omitted, and shows that normalization is crucial, at least for Ss-LrpB and Sa-LysM, the proteins studied in these datasets. Furthermore, these experiments show that smear is often the most important remaining error source, while background noise variations are in general comparatively small. The obtained high accuracies have allowed us to reliably determine cooperative binding constants for Ss-LrpB [11], and to derive a position weight matrix for Sa-LysM that could accurately predict binding motifs in regions detected by ChIP-chip analysis [5].
